# In-Season Repeated-Sprint Training in Hypoxia in International Field Hockey Players

**DOI:** 10.3389/fspor.2020.00066

**Published:** 2020-07-08

**Authors:** Carl James, Olivier Girard

**Affiliations:** ^1^Institut Sukan Negara (National Sports Institute), Kuala Lumpur, Malaysia; ^2^School of Human Sciences (Exercise and Sport Science), The University of Western Australia, Crawley, WA, Australia

**Keywords:** repeated-sprint training in hypoxia, repeated-sprint ability, team sports, hockey, sprint performance

## Abstract

Repeated-sprint training in hypoxia (RSH) studies conducted “*in-season”* are scarce. This study investigated the effect of discontinuous, running-based RSH, on repeated-sprint treadmill performance in hypoxia in a team sport cohort, prior to international competition. Over a 6-week “*in-season*” period, 11 elite male players (Malaysia national team) completed eight multi-set RSH sessions on a non-motorized treadmill in a normobaric hypoxic chamber (FiO_2_ = 13.8%). Three testing sessions (Sessions 1, 5, and 8), involved three sets of 5 × 8-s sprints, with 52-s recovery between sprints and 4–5 min between sets. Training sessions (Sessions 2, 3, 4, 6, and 7) consisted of four to five sets of 4–5 × 8-s sprints. During testing sessions, maximum sprinting speed was recorded for each sprint with values averaged for each set. For each set, a peak speed and fatigue index were calculated. Data were compared using two-way repeated measures ANOVA (sessions × sets). Average speed per set increased between testing sessions (*p* = 0.001, $\eta_{\text{p}}^{2}$ = 0.49), with higher values in Session 8 (25.1 ± 0.9 km.h^−1^, +4 ± 3%, *p* = 0.005), but not Session 5 (24.8 ± 1.0 km.h^−1^, +3 ± 3%, *p* = 0.405), vs. Session 1 (24.2 ± 1.5 km.h^−1^). Peak sprinting speed in each set also increased across testing sessions (*p* = 0.008, $\eta_{\text{p}}^{2}$ = 0.382), with Session 8 (26.5 ± 1.1 km.h^−1^) higher than Session 5 (25.8 ± 1.0 km.h^−1^, +1 ± 4%, *p* = 0.06) and Session 1 (25.7 ± 1.5 km.h^−1^, +4 ± 4%, *p* = 0.034). Fatigue index differed between sessions (*p* = 0.04, $\eta_{\text{p}}^{2}$ = 0.331, Session 1; −6.8 ± 4.8%, Session 5; −3.8 ± 2%, Session 8; −5.3 ± 2.6%). In international field hockey players, a 6-week in-season RSH program improved average and peak, repeated treadmill sprint speeds following eight, but not five sessions.

## Introduction

Field hockey is a team sport that features in major international competitions such as the Commonwealth and Olympic Games. As in other field-based team sports such as soccer (Buchheit et al., 2010) and rugby league (Gabbett and Wheeler, 2015), the ability to complete repeated bouts of sprinting with short recovery times would be advantageous in field hockey (Spencer et al., 2004b; Lythe and Kilding, 2011). Previously, average total running distance per game has been reported as >9,000 m, of which >2,000 m is high-speed running (>4.17 m.s^−1^) (Jennings et al., 2012). Over the past decade, the physical demands of international hockey have evolved, reflecting the introduction of “faster” synthetic pitches and various rule changes, which target “high intensity” matches and fewer breaks in play (International Hockey Federation, 2019). Notable rule changes include unrestricted “rolling” substitutions during games, quick restarts and the game structure changing to four, 15-min quarters, rather than two 35-min halves. Such changes have resulted in matches characterized by a lower total distance, but greater proportion of high-speed running (Ihsan et al., 2018; McMahon and Kennedy, 2019). From a tactical perspective, coaches take advantage of additional substitution opportunities to maximize the number of on-field, high-intensity displacements at the team level (Linke and Lames, 2017). Therefore, while the importance of repeated-sprint ability for hockey performance has long been recognized (Spencer et al., 2004a; Lythe and Kilding, 2011), implementation of recent rule changes accentuates the importance of this fitness component.

The international playing schedule typically provides “in-season” preparation periods of only 2–3 weeks between tournaments. Support teams therefore consistently seek time-efficient training strategies, allowing concomitant preservation of sport-specific priorities, while achieving physiological development of their players. The optimal training to improve repeated-sprint ability remains equivocal, reflecting the complex integration of metabolic and/or neuromuscular regulatory systems that, in turn, dictate on-field physical performance (Bishop et al., 2011). Repeated-sprint training in a hypoxic environment (RSH) involves the repetition of maximal, short-duration (<30 s) efforts, interspersed with incomplete recoveries (Faiss et al., 2013). An emerging body of evidence indicates that RSH provides an additive performance benefit compared with the normoxic equivalent (Brocherie et al., 2017a). While traditional altitude training approaches necessitate a prolonged period at altitude, RSH is a new addition to the “live low–train high” (LLTH) paradigm (Millet et al., 2013). Despite the apparent popularity of this training method, with more than 25 studies now published since 2013 (Millet et al., 2019), the majority of literature has utilized cycling exercise, with moderately trained participants, outside of a competitive season. To date, there is a dearth of evidence demonstrating the effectiveness of RSH when implemented with elite athletes, from running-based team-sports, during a competitive season.

For field hockey players, implementing running-based repeated-sprint training is advantageous for training specificity. It has previously been highlighted that, compared with cycling protocols, running sessions may benefit from a greater oxidative and metabolic strain (Girard et al., 2013). Consequently, for team sport athletes, a performance benefit from cycling RSH may not be as direct as for running (Goods et al., 2015). Running RSH protocols can be performed using portable inflatable hypoxic marquees, which permit over-ground running-based RSH training (Girard et al., 2013). Reportedly, six repeated-sprint sessions (4 × 5 × 5-s maximal sprints) performed at FiO_2_ ~ 14.5%, can boost repeated-sprint performance and perceptual responses in international field hockey players (Brocherie et al., 2015b, 2017b). However, such equipment remains expensive. Moreover, while running protocols provide specificity, implementing additional over-ground running training during the competitive phase (in-season) is challenging, without unduly increasing the overall training load (Goods et al., 2015). Successful RSH implementation that uses a running protocol may therefore require less frequent sessions and/or discontinuous training prescription, in order to effectively manage sprinting loads alongside other training priorities. Furthermore, sprinting on a non-motorized treadmill (NMT) installed in a hypoxic chamber may be an alternative approach to over-ground running at real or simulated altitude (i.e., hypoxic marquee). NMTs permit maximal sprinting and, unlike a motorized treadmill, include a representative acceleration component, while also demonstrating higher cardiometabolic demands (Edwards et al., 2017). Together, this may accentuate physiological strain in a manner complementary to hypoxia, while moderating external loads and permitting unrestricted maximal sprinting during training.

Therefore, the aim of this study was to provide a thorough description of eight sessions of a running-based RSH protocol on an NMT, integrated within the overall preparation plan over a 6-week training period, during the lead-up to a major international competition. We detail how RSH may be implemented when many elements compete for a player's time, in a real-world context, that involves international fixtures and long-haul travel. Given the discontinuous nature of our RSH prescription, these data will form the basis for discussing how RSH sessions can be practically suited to the modern-day team. We hypothesized that RSH would elicit early (i.e., Session 5) and late (i.e., Session 8) improvements in repeated-sprint performance in hypoxia for international field hockey players.

## Methods

### Participants

Eleven international field hockey players (age 26.1 ± 4.2 years, stature 170 ± 5 cm, body mass 64.6 ± 4.8 kg, sum of 7 skinfolds 44.2 ± 10.4 mm) participated as part of their training in preparation for a major international competition. The training group comprised four defenders, four midfielders, and three forwards. All players followed the same training/recovery program and had been training full time with the Malaysia national team for >9 months (world ranking #11). This protocol was conducted in accordance with the Code of Ethics of the World Medical Association (Declaration of Helsinki). Ethical approval was not sought as these training sessions occurred as part of player's routine training with the national team, as part of their employment, with data retrospectively analyzed (Winter and Maughan, 2009).

### Design

Over a 6-week “in-season” period, players completed eight repeated-sprint training sessions on an NMT (Woodway Curve, Woodway USA Inc, Waukesha, WI, USA) in a normobaric hypoxic chamber (Welltech Instruments, Hong Kong). Where possible, two training sessions per week were scheduled, integrated with other training requirements, competitive matches and travel. For all RSH sessions, environmental conditions were set to simulate an altitude of 3,000 m (F_I_O_2_ = 13.8%) and thermostatically controlled at an ambient temperature of 21°C and 45% relative humidity. Session 1 was replicated during Session 5 and Session 8, in order to assess training effectiveness.

During the 6-week period, other trainings included 17 on-field Hockey sessions, 10 strength sessions, 4 international hockey matches and 1 recovery session (Table 1). Prior to the training block, players competed in an international tournament, which involved four matches in 5 days. Players then completed a week of decentralized, active recovery, encompassing 2 × 30-min runs and two strength sessions.

**Table 1:** Overview of the sessions and mean (± SD) of training load within 6-week training block.

		**Mon**	**Tue**	**Wed**	**Thu**	**Fri**	**Sat**	**Sun**	Total training time (min)*	***Playerload (a.u.)***	Number of sprints**	Sprinting distance (m)***
Week 1	AM		RSH		Strength	RSH	Strength		242 ± 28	1,464 ± 290	77 ± 14	1,416 ± 243
	PM		Strength	Hockey			Hockey					
Week 2	AM	Hockey	RSH	Strength	RSH	Strength			448 ± 5	2,242 ± 185	97 ± 12	1,612 ± 209
	PM	Strength	Hockey			Hockey						
Week 3	AM	Strength	RSH		Travel	Recovery	Hockey		372 ± 16	2,408 ± 153	91 ± 2	1,523 ± 52
	PM	Hockey	Hockey				Hockey	Hockey				
Week 4	AM	International match	International match	Hockey	International match	International match		Travel	241 ± 23	2,159 ± 46	89 ± 8	1,699 ± 161
	PM											
Week 5	AM				Hockey	Strength	Hockey		219 ± 10	1,316 ± 112	41 ± 3	765 ± 88
	PM				Hockey	RSH						
Week 6	AM	RSH	Strength	Hockey	RSH	Strength			306 ± 24	1,922 ± 158	71 ± 7	1,290 ± 113
	PM	Hockey	Hockey		Hockey							

* *Training time represents the sum of repeated sprinting in hypoxia (RSH), field hockey and strength training sessions. Strength training sessions are included with a standardized duration of 60 min. Training time does not include warm-up or cool downs*.

** *Number of sprints includes field hockey and RSH sprints*.

*** *Sprinting distance represents GPS-derived distance from field hockey training combined with standardized distance per RSH sprinting effort*.

### Repeated-Sprint Training in Hypoxia

A multi-set RSH protocol was implemented, in order to induce significant fatigue levels, but avoiding the use of pacing strategies. Our work: rest ratio was similar to relevant field hockey literature (Brocherie et al., 2017b), and sprint durations were in keeping with what would be necessary to achieve maximum speed on an NMT (Brown et al., 2017). Planned progression was achieved by manipulating the number of repetitions (reps) within each set, the total number of sprints and/or the rest period between sets (Table 2). This was modified as necessary, through proactive training load management, e.g., reduced reps if weekly sprinting meters during field training exceeded planned stimulus. Where a training progression was planned, but running training loads were considered to be high, either rest periods between sets were reduced to enhance internal strain or session structure was altered to include more reps per set, while maintaining the same total number (e.g., 4 × 5 instead of 5 × 4). All sessions involved between 15 and 20 sprints, with a maximum of 5 sprints per set and included 4- to 5-in rest periods between sets.

**Table 2:** Overview of repeated sprint training in hypoxia sessions.

**Session**	**Work (s)**	**Rest between**	**Sets**	**Reps**	**Total**	**Rest between**
		**reps (s)**			**sprints**	**sets (min)**
1*	8	52	3	5	15	00:05:00
2	8	52	4	4	16	00:05:00
3	8	52	4	4	16	00:05:00
4	8	52	5	4	20	00:04:00
5*	8	52	3	5	15	00:04:00
6	8	52	4	5	20	00:05:00
7	8	52	4	4	16	00:05:00
8*	8	52	3	5	15	00:05:00

All sessions were completed on a non-motorized treadmill in hypoxia (FiO_2_ = 13.8%) with passive rest. *Testing sessions (Sessions 1, 5, and 8).

Prior to every session, players undertook a self-guided 15-min outdoor warm-up, which included low-intensity jogging and specific mobility/activation exercises. Upon entering the chamber, within 2 min, players completed three standardized sub-maximal efforts at speeds corresponding to 15, 19, and 22 km.h^−1^ on the NMT. Players performed maximal accelerations until the respective target speed and then maintained this for 5 s. All players had considerable prior experience of sprinting on the NMT from previous training.

Before each sprint, players were given a countdown and specific verbal cues. These clues included to deliver a maximum effort, to focus only on that sprint and not to employ any pacing strategy. Players began each sprint from a standing position in the center of the treadmill belt and were not permitted to hold onto the side rails to “drive” the belt forward. Moreover, players were told to sprint maximally until they heard “stop” after 8 s (Brown et al., 2017), at which point they jumped astride of the NMT belt, and the 52-s rest began. All rest between sets was passive, with players standing away from the treadmill, within the chamber. Practically, three players worked in a 1-min rotation, with 20 s per player to get on the NMT, perform the sprint, and get off the NMT. The peak speed on every sprint was noted. On average, this occurred around 6–7 s into the sprint. Players were told their maximum speed after each sprint for motivation and encouraged to exceed this on each effort. After the final sprint, participants remained in the chamber for the subsequent 5-min rest period.

During RSH sessions, players wore a 100-Hz accelerometer/GPS unit (G5, Catapult Sports, Melbourne, Australia) harnessed between the scapulae in a customized sports vest (Barrett et al., 2014). Players wore the sports vest from the start of the warm-up until completion of the warm-down, although the activity was cropped to only include sprint data. Each player had worn the vest previously and used the same unit throughout the training period, including for field sessions. Accelerometer data was downloaded with associated software (Openfield, Catapult Sports, Melbourne, Australia) to derive *Playerload*. *Playerload* is the square root of the sum of the squared instantaneous rate of change in acceleration in the x, y, and z axes, divided by 100, and expressed in arbitrary units (Cardinale and Varley, 2017). The coefficient of variation of *Playerload* has been reported as 5.9% across a range of treadmill speeds (Barrett et al., 2014).

### Repeated-Sprint Testing Sessions

Three standardized training sessions were used to assess RSH effectiveness. Sessions 1, 5, and 8 involved running three sets of five sprints on an NMT. Each sprint lasted 8 s, followed by 52 s of rest. The rest period between sets was 5 min for sessions 1 and 8, and 4 min during session 5. The rest period during session 5 was shorter because a progression of the training stimulus was planned that did not result in increased running loads. The peak speed achieved during each sprint was noted. Fatigue index was calculated as a percentage decrement for each set, in accordance with Glaister et al. (2008). All testing sessions occurred at the same time of the day, although no taper period was planned before any testing session, reflecting the “in-season” nature of the intervention.

### Statistical Analysis

All outcome variables were assessed for normality and sphericity prior to further analysis. Performance outcomes such as average speed, peak speed, and fatigue index during sessions 1, 5, and 8 were analyzed using two-way repeated measures ANOVA. The average of each set of five sprints was entered into the ANOVA to identify differences between sets (within session) and between testing sessions. In light of the discontinuous prescription of RSH around international fixtures, a further two-way repeated measures ANOVA was completed on four sprints from the first three sets of sessions 1, 3, 6, and 8. Average speed per set and the fatigue index for each set were similarly entered into the ANOVA. Bonferroni correction was applied during *post hoc* analysis where significant differences were identified. The total *Playerload* from all sprints completed during testing sessions were compared using paired samples t-tests. Data were analyzed using SPSS (Version 25, SPSS Inc, IL, USA) with statistical significance set at *p* < 0.05 and data presented as mean ± SD. Effect sizes for main effects and interaction effects are presented as partial eta squared (partial η^2^).

## Results

The average RSH *Playerload* (139 ± 24 a.u.) represented 31% of the average Match *Playerload* (452 ± 20 a.u.) and 32% of the average Hockey training *Playerload* (429 ± 124 a.u.), within this training period (Table 1). Across the 6-weeks, RSH *Playerload* represented 10% of the total *Playerload* (Week 1: 18%, Week 2: 14%, Week 3: 5%, Week 4: 0%, Week 5: 13%, and Week 6: 13%).

### Comparison of Testing Sessions

**Figure 1:**
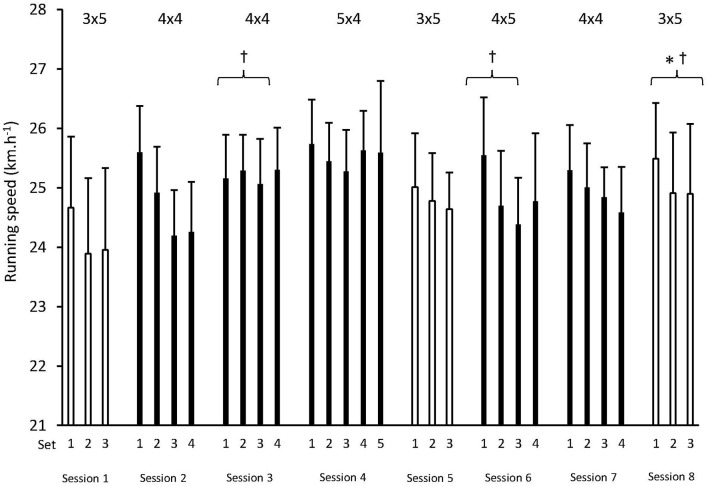
Mean ± SD average running speeds of each set during every training session. Testing sessions (1, 5, and 8) are shown as white columns. *Statistical difference compared to testing sessions 1 and 5 (*p* < *0.05*). ^†^Statistical difference compared to session 1, when the first four sprints of three sets are analyzed (sessions 1, 3, 6, and 8 only were entered into analysis). Session structure (sets × reps) is displayed above the respective columns.

There was a main effect for a higher average set speed across testing sessions (*p* = 0.001, $\eta_{\text{p}}^{2}$ = 0.489). The average speeds for each set are shown in Figure 1. Pairwise comparisons revealed higher speeds during Session 8 (25.1 ± 0.9 km.h^−1^, +4 ± 3%, *p* = 0.005), but not during Session 5 (24.8 ± 1 km.h^−1^, +3 ± 3%, *p* = 0.405), compared to Session 1 (24.2 ± 1.5 km.h^−1^). A statistically significant difference was not observed between Session 1 and Session 5 (*p* = 0.086). A main effect was also observed for average set speed between sets within testing sessions (*p* = 0.029, $\eta_{\text{p}}^{2}$ = 0.297). Pairwise comparisons revealed a drop in speed from Set 1 (25.1 ± 1 km.h^−1^) to Set 2 (24.5 ± 1.3 km.h^−1^, −2 ± 4%, *p* = 0.005), but not between Set 1 and Set 3 (24.5 ± 1.2 km.h^−1^, −1 ± 6%, *p* = 0.119) or between Set 2 and Set 3 (*p* = 1.000). No interaction effect (sessions × sets) was observed (*p* = 0.866, $\eta_{\text{p}}^{2}$ = 0.031).

There was a main effect for increased peak sprinting speed across testing sessions (*p* = 0.008, $\eta_{\text{p}}^{2}$ = 0.382). Pairwise comparisons revealed that peak speed during sets 1, 2, and 3 of Session 8 (26.5 ± 1.1 km.h^−1^) was higher than during Session 1 (25.7 ± 1.5 km.h^−1^, +4 ± 4%, *p* = 0.034) and higher than Session 5 (25.8 ± 1 km.h^−1^, 1 ± 4%, *p* = 0.06). A main effect was also observed for peak speed between sets within testing sessions (*p* = 0.029, $\eta_{\text{p}}^{2}$ = 0.298). However, pairwise comparisons did not reveal statistically significant differences between Set 1 (26.3 ± 1 km.h^−1^), Set 2 (25.8 ± 1.4 km.h^−1^), or Set 3 (25.8 ± 1.2 km.h^−1^). No interaction (sessions × sets) was observed (*p* = 0.822, $\eta_{\text{p}}^{2}$ = 0.037).

There was a main effect for fatigue index within each set, between testing sessions (*p* = 0.04, $\eta_{\text{p}}^{2}$ = 0.331). The largest fatigue index was observed in session 1 (−6.8 ± 4.8%), followed by session 8 (−5.3 ± 2.6%, *p* = 0.822 vs. session 1), and the smallest value in session 5 (−3.8 ± 2%, *p* = 0.088 vs. session 1). Fatigue index did not differ between sessions 5 and 8 (*p* = 0.133). There was no main effect for fatigue index across sets within testing sessions (*p* = 0.448, $\eta_{\text{p}}^{2}$ = 0.095) or any interaction between sessions and sets (*p* = 0.087, $\eta_{\text{p}}^{2}$ = 0.218).

**Figure 2:**
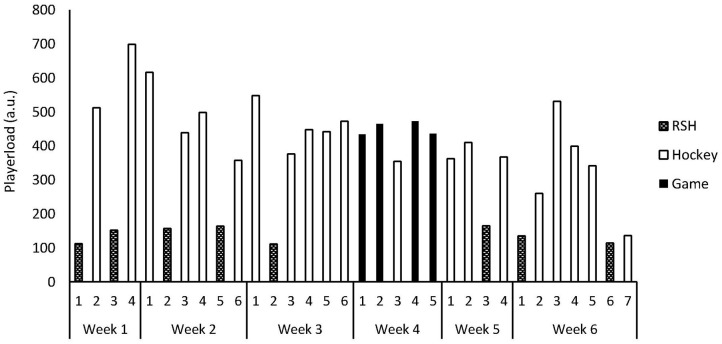
Squad average *Playerload* load across the 6-week training period. Activities are categorized as repeated sprinting in hypoxia (RSH), on-field hockey training (Hockey), and international hockey matches (Game).

*Playerload* did not differ between Session 1 (112 ± 38 a.u.), Session 5 (111 ± 12 a.u.), and Session 8 (114 ± 16 a.u.; *p* = 0.667; Figure 2).

### Analysis of Testing and Training Sessions

Upon analyzing the first four sprints of sessions 1, 3, 6, and 8, there was a main effect for running speed across training sessions (*p* = 0.001, $\eta_{\text{p}}^{2}$ = 0.456). Pairwise comparisons revealed higher speeds during session 3 (25.0 ± 1.1 km.h^−1^, +4 ± 5%, *p* = 0.009), session 6 (24.9 ± 1.2 km.h^−1^, +4 ± 5%, *p* = 0.016), and session 8 (24.9 ± 1.1 km.h^−1^, +4 ± 5%, *p* = 0.014), compared with session 1 (24.1 ± 1.6 km.h^−1^). No other differences between sessions were observed (all *p* > 0.05). A main effect was also observed for differences in average speed across sets, within sessions (*p* = 0.002, $\eta_{\text{p}}^{2}$ = 0.424). Pairwise comparisons revealed a drop in speed from set 1 (25.2 ± 1 km.h^−1^) to set 2 (24.6 ± 1.4 km.h^−1^, −2 ± 4%, *p* = 0.025) and from set 1 to set 3 (24.4 ± 1.4 km.h^−1^, −3 ± 5%, *p* = 0.023), but not between set 2 and set 3 (*p* = 0.763). The sessions × sets interaction effect for average set speed was not significant (*p* = 0.078, $\eta_{\text{p}}^{2}$ = 0.154).

Across these four sessions, a main effect for fatigue index was observed (*p* = 0.001, $\eta_{\text{p}}^{2}$ = 0.424), with the largest value observed in session 1 (−6.3 ± 4.2%), followed by session 8 (−4.6 ± 2.4%), session 6 (−4.3 ± 2.3%) and session 3 (−3.4 ± 2.2%). However, the only significant difference between sessions was observed between sessions 1 and 3 (*p* = 0.005). There was no main effect of fatigue index across sets (*p* = 0.186, $\eta_{\text{p}}^{2}$ = 0.155) or an interaction effect between sessions and sets (*p* = 0.073, $\eta_{\text{p}}^{2}$ = 0.170).

## Discussion

We report the effect of eight, running RSH sessions, integrated alongside existing field-hockey training, on repeated-sprint performance in hypoxia across a 6-week, in-season period. Significant increases in both the set average and peak speed, as well as a lower fatigue index, between testing sessions 1 and 8 were observed, while changes between testing sessions 1 and 5 were not significant. Further analysis also revealed improved average set speed and a lower fatigue index from the third session. A discontinuous, running-based RSH block, spread across 6 weeks, appears beneficial when implemented within the competitive season in international field-hockey players, with RSH sessions representing on average ~10% (range: 0–18%) of weekly total training load.

Our data support an emerging body of research pertaining to RSH as an effective training modality for team sports (Galvin et al., 2013; Brocherie et al., 2015a; Hamlin et al., 2017; Brechbuhl et al., 2018). Unlike most existing research, we did not implement RSH as an isolated intervention, nor were we able to utilize a control training group. However, our data indicate that RSH can be integrated with sport-specific training to elicit repeated-sprint improvements in elite athletes when incorporated in-season, in a real-life environment (Brocherie et al., 2017b; Beard et al., 2019a,b). Previously, Brocherie et al. (2015b) implemented six RSH sessions (over-ground running) during a 14-day “live high, train low” training camp, with elite field hockey players. Using a similar multi-set structure (four sets of 5 × 5-s sprints) to the present study, the authors reported twice larger performance gains (+3.7 vs. 1.9%) relative to the normoxic equivalent when eight, 20-m sprints were repeated. These benefits, as well as increased hemoglobin mass and endurance performance, remained for 3 weeks post-intervention in the RSH group only. However, the “live high” aspect of this study resulted in a greater hypoxic exposure than our “live low” RSH sessions; therefore, these effects cannot be solely attributed to RSH. In elite rugby players, Beard et al. (2019a) reported improvements in repeated power production following just four cycle-based, multi-set RSH sessions (three sets of 8 × 10-s sprints). In our testing sessions involving three sets of five sprints, we observed statistical differences only after eight sessions, rather than five, but the density of RSH sessions was lower than that of Brocherie et al. (2015b), due to the discontinuous nature of our in-season RSH training prescription. While our data from a running-based RSH protocol appear to contrast the fast, cycling induced improvements reported by Beard et al. (2019a) from four RSH sessions completed within 2 weeks, this may be influenced by our study design. For example, our mid-point assessment during Session 5 did not have a pre-test taper (Table 1) and involved shorter rest periods between sets, compared with Session 1 (4 min vs. 5 min). This reflected a planned progression of the training stimulus, while managing overall training volume, given that four competitive matches were scheduled for the following week. Therefore, the findings of session 5 should be interpreted within the context of slightly shortened rest period, but still have merit, given the matched session structure and volume (three sets of five sprints). Our secondary analysis of training data revealed improved average speed and a smaller fatigue index from session 3, thereby reinforcing RSH benefits. We also highlight that our testing sessions were performed within hypoxia, which is in contrast to much of the related literature (Brocherie et al., 2017b; Beard et al., 2019a,b). This may result in a different magnitude of improvement than if assessments were made in normoxia. Future research should consider the optimal dose, at both session and mesocycle level, in order to identify the most effective periodization of this intervention.

On average, RSH represented 10% of the weekly training volume (Figure 2), which is supportive of RSH being a time-efficient training modality. The timing of this study required sessions to be planned around other training priorities, such as practice matches. Therefore, players would not be considered “rested” during testing Sessions 5 and 8. Furthermore, the training intervention had 1-week (Week 4) with no RSH sessions and long-haul international travel. Notwithstanding, RSH still revealed an improvement of 4% (+1 km.h^−1^) in average speed during Session 8. This would appear a practically meaningful effect, as all completed sprints exceeded 19 km.h^−1^, which is commonly used as a sprinting threshold within field hockey (Macutkiewicz and Sunderland, 2011). Furthermore, this improvement exceeds the previously reported reliability of this model of NMT during a 6-s sprint at comparable (~25 km.h^−1^) speeds (coefficient of variation of 1.3%; Sirotic and Coutts, 2008). Given the moderate duration of exposure to hypoxia that RSH delivers, improvements are likely due to skeletal muscle, rather than hematic adaptations (Wilber et al., 2007), in turn, increasing fatigue resistance of fast-twitch fibers (Faiss et al., 2013). However, we do not have suitable physiological measures to verify this suggestion within our cohort.

Previous RSH studies of international hockey players utilized an inflatable hypoxic marquee, which permitted 40-m over-ground sprinting (Brocherie et al., 2015b, 2017b). This reflects the apparent importance of RSH specificity, as reported by Goods et al. (2015). Despite this, these authors also highlighted potential drawbacks of running RSH, given the need for effective load management when implementing running RSH protocols. For example, field sessions may have to be modified to accommodate RSH without overloading players (Goods et al., 2015). However, our encouraging findings indicate that RSH can be effectively integrated in-season, when repeated-sprint training would already be a training focus within the periodized plan. As with a previous running-based study (Girard et al., 2017), our testing sessions revealed a decrease in running speeds from set 1 to set 2, but not between sets 2 and 3. This confirms that early and large performance decrements occur during repeated-sprint exercise that use a multi-set approach, with peripheral disturbances being presumably the main cause of fatigue (Collins et al., 2018). Future research may consider whether greater benefits can be derived from RSH if the work:rest ratio is individualized, which requires identification of an optimal trade-off between maintaining training quality (i.e., attaining close to maximum sprinting speed) and maximizing physiological strain.

Our choice of treadmill may also provide benefits for managing player's loads. We utilized a curved NMT for training, as has previously been used for similar running RSH protocols (Morrison et al., 2015a,b). Such treadmills facilitate maximal sprinting, along with representative accelerations and decelerations, without the need to wear a harness. Similar, flat-belt NMTs present lower running speeds compared with over-ground running, but acceleration and velocity metrics are correlated with radar guns (Morin and Sève, 2011) and timing gates (Highton et al., 2012). A greater cardiometabolic strain is also observed on a curved NMT for a given speed, compared with motorized treadmills or over-ground running, reflecting the inertia associated with the weight of the belt (Edwards et al., 2017). Consequently, within our field hockey cohort, an NMT appears beneficial as it served to maintain internal load, for a given or potentially reduced, external load (Impellizzeri et al., 2019). In turn, this makes running-based RSH protocols easier to implement in-season. Anecdotally, players did not feel that RSH sessions elicited comparable soreness to some field sessions. The greater cardiometabolic strain observed when using curved NMTs may reflect the need to run closer to the front of the belt in order to accelerate maximally. This is akin to hill runs, given that the belt may be 5–10° above horizontal at the front. Therefore, running mechanics may be altered and an enhancing energy cost observed during curved NMT sprinting in a manner similar to a motorized treadmill gradient or hill sprints (Vernillo et al., 2017).

While the absence of a control training group inhibits interpretation of the independent effect of hypoxia within our cohort, mounting evidence indicates a greater benefit from RSH compared with repeated-sprint training at sea level (Brocherie et al., 2017a). In the current study, the overall physical training program was modified to integrate RSH while providing consistent total weekly sprinting meters. For example, on-field hockey and specific speed drills that involved significant sprinting distances were reduced during these 6-weeks to avoid additional sprinting to what the players were accustomed to (~1,500 m per week). Therefore, the sprinting volume the players covered during the 6-wk training block (Table 1) was in keeping with prior training weeks, which is indicative of a training effect following RSH. While there was an apparent reduction in load during week 5, this week may not be accurately construed as rest, given it involved considerable long-haul travel and sleep loss. Moreover, analysis of training data from training sessions 3 and 6, nor sessions 6 and 8, revealed a difference. This indicates that the apparent reduced load during week 5 did not have a beneficial effect. Notwithstanding, we cannot discount that the hockey training and matches that were concurrent to RSH had a positive impact on performance results during Session 8 and to a lesser extent on that of Session 5, compared with that of Session 1.

Although a curved NMT offers a convenient method for team sport athletes to utilize RSH, we observed considerable variability in running speeds between sessions, as shown by the large standard deviations in Figure 1. This was in spite of apparent consistent maximal efforts. Anecdotally, some players indicated they had difficulty to “efficiently accelerate the belt” under fatigue. However, this model of NMT has previously been shown to be both valid and reliable across a range of representative team-sport speeds, including sprinting (Sirotic and Coutts, 2008; Aldous et al., 2014; Tofari et al., 2014). The lack of any difference in *Playerload* between testing sessions indicates that there was no learning effect or changes in gait as players completed more sessions on the curved NMT, although we recognize a lack of validity data specifically pertaining to *Playerload* on a curved NMT. Our data do not reveal clear trends in terms of average speed reduction or fatigue index between or within sets. Therefore, such variability is likely to be attributable to individual fatigue resulting from the accumulated training load during this period. Notwithstanding this variability, alongside our average improvement of 4%, 9 out of 11 players displayed improvements greater than 2% during Sessions 1 to 8.

### Practical Applications

This study builds on recent literature demonstrating RSH to be a time-efficient training method for improving repeated-sprint performance in elite athletes in “real world” scenarios (Beard et al., 2019a,b). Moreover, these data demonstrate performance is improved when repeated sprinting is assessed in hypoxia. Eight RSH sessions, across a 6-week in-season period, were effectively integrated into the overall physical training program, which included a 1-week period of travel and international matches, providing a repeated-sprint performance benefit.

## Data Availability Statement

The datasets generated for this study are available on request to the corresponding author.

## Ethics Statement

This protocol was conducted in accordance with the Code of Ethics of the World Medical Association (Declaration of Helsinki). Ethical approval was not sought as these training sessions occurred as part of player's routine training with the national team, as part of their employment, with data retrospectively analyzed (Winter and Maughan, 2009).

## Author Contributions

CJ and OG conceived and designed the study, analyzed and interpreted the data, drafted and revised the manuscript, and approved the final version of the manuscript. CJ collected the data. All authors contributed to the article and approved the submitted version.

## Conflict of Interest

The authors declare that the research was conducted in the absence of any commercial or financial relationships that could be construed as a potential conflict of interest. The reviewer MH declared a past co-authorship with one of the authors CJ to the handling editor.
